# Linked candidate genes of different functions for white mold resistance in common bean (*Phaseolus vulgaris* L) are identified by multiple QTL mapping approaches

**DOI:** 10.3389/fpls.2023.1233285

**Published:** 2023-07-31

**Authors:** Atena Oladzad, Jayanta Roy, Sujan Mamidi, Phillip N. Miklas, Rian Lee, Josh Clevenger, Zachary Myers, Walid Korani, Phillip E. McClean

**Affiliations:** ^1^ Genomics Data Scientist II, Sound Agriculture, Emeryville, CA, United States; ^2^ Department of Plant Sciences, North Dakota State University, Fargo, ND, United States; ^3^ Hudson Alpha Institute for Biotechnology, Huntsville, AL, United States; ^4^ Grain Legume Genetics and Physiology Research Unit, United States Department of Agriculture - Agricultural Research Service (USDA-ARS), Prosser, WA, United States; ^5^ Genomics, Phenomics, and Bioinformatics Program, North Dakota State University, Fargo, ND, United States

**Keywords:** white mold, common bean, linkage mapping, bulk-segregant analysis, QTL-seq, genomic regions, candidate gene, marker-assisted selection

## Abstract

White mold (WM) is a major disease in common bean (*Phaseolus vulgaris* L.), and its complex quantitative genetic control limits the development of WM resistant cultivars. WM2.2, one of the nine meta-QTL with a major effect on WM tolerance, explains up to 35% of the phenotypic variation and was previously mapped to a large genomic interval on Pv02. Our objective was to narrow the interval of this QTL using combined approach of classic QTL mapping and QTL-based bulk segregant analysis (BSA), and confirming those results with Khufu *de novo* QTL-seq. The phenotypic and genotypic data from two RIL populations, ‘Raven’/I9365-31 (R31) and ‘AN–37’/PS02–029C–20 (Z0726-9), were used to select resistant and susceptible lines to generate subpopulations for bulk DNA sequencing. The QTL physical interval was determined by considering overlapping interval of the identified QTL or peak region in both populations by three independent QTL mapping analyses. Our findings revealed that meta-QTL WM2.2 consists of three regions, WM2.2a (4.27-5.76 Mb; euchromatic), WM 2.2b (12.19 to 17.61 Mb; heterochromatic), and WM2.2c (23.01-25.74 Mb; heterochromatic) found in both populations. Gene models encoding for gibberellin 2-oxidase 8, pentatricopeptide repeat, and heat-shock proteins are the likely candidate genes associated with WM2.2a resistance. A TIR-NBS-LRR class of disease resistance protein (Phvul.002G09200) and LRR domain containing family proteins are potential candidate genes associated with WM2.2b resistance. Nine gene models encoding disease resistance protein [pathogenesis-related thaumatin superfamily protein and disease resistance-responsive (dirigent-like protein) family protein etc] found within the WM2.2c QTL interval are putative candidate genes. WM2.2a region is most likely associated with avoidance mechanisms while WM2.2b and WM2.2c regions trigger physiological resistance based on putative candidate genes.

## Introduction

White mold (WM), caused by *Sclerotinia sclerotiorum* (Lib.) de Bary, is a ubiquitous destructive fungal disease mostly prevalent in cool, moist conditions. Depending on the common bean genotype, it can cause from 30% to 100% yield losses (Schwartz and Singh, 2013). All the aerial parts of the plant can be infected by this disease. A white, cottony appearance of mycelium growing on the surface of pods, leaves, branches, and stems is a visual characteristic of this disease. As symptoms progress, water-soaking followed by desiccation of the affected tissue leads to a dried bleached appearance of stems and branches (Schwartz and Singh, 2013; Singh et al., 2014). Under field conditions, *S. sclerotiorum* acts as a hemi-biotroph, first using biotrophic infection processes and later necrotrophic mechanisms to invade its host. In the biotrophic period, the pathogen uses living tissues of the plant as a source of nutrition and suppresses the host immune system. Eventually, the pathogen kills host tissue extensively and uses the dead cells as nutrients to progress its life-cycle (Kabbage et al., 2015). The long-term survival of *S. sclerotiorum* is mainly via sclerotia (hardened mycelium) formation. These structures are formed on or within infected plants during the necrotrophic period and deposited in the soil, allowing the fungus to survive for years until environmental conditions become favorable (Schwartz and Steadman, 1989; Miklas et al., 2013).

Complete resistance to this disease does not exist in commercial common bean cultivars (Miklas and Grafton, 1992; Miklas et al., 2001; Singh and Schwartz, 2010; Vasconcellos et al., 2017). Partial resistance, available in some common bean materials, is associated with avoidance or physiological resistance mechanisms, or a combination of both mechanisms. Architectural features such as canopy density, canopy height, and lodging resistance (Balasubramanian et al., 2013; Miklas et al., 2013) influence avoidance mechanisms. Physiological resistance involves plant defense against pathogen effectors (Mamidi et al., 2016).

The complex, quantitative genetic control of both avoidance and physiological mechanisms, along with environmental effects, has limited the development of WM resistance in common bean. Although conventional breeding has provided a limited number of lines with partial resistance, there is a need for more effective tools for routine generation of lines with high levels of WM resistance in common bean. Identification of QTL or genomic regions associated with traits of interest provides the basis for the development of functional molecular markers to implement marker-assisted selection (MAS). The MAS for WM resistance would be beneficial, as selections could be made in the early stages of the breeding process with less reliance on phenotyping large number of samples in the presence of the pathogen which will accelerate the breeding process and would be cost-effective. Recently, Vasconcellos et al. (2017) presented a meta-analysis that identified 37 QTL associated with partial WM resistance across multiple populations and environments. These QTL were identified using traditional QTL analyses and clustered into nine meta-QTL: WM1.1, WM2.2, WM3.1, WM5.4, WM6.2, WM7.1, WM7.4, WM7.5, and WM8.3. These meta-QTL had significant effects across multiple environments, and the QTL came from different genetic backgrounds. In most cases the QTL region intervals were megabases in width, making it difficult to identify candidate genes. Among these nine meta-QTL, the highest percentage of phenotypic variation was explained by WM2.2 located within the 4.54–22.98 Mb interval on chromosome Pv02 (Vasconcellos et al., 2017). This QTL was reported in at least six populations and explained up to 35% of the phenotypic variation for disease reaction in both field (avoidance and physiological mechanisms) and greenhouse (physiological mechanisms) tests (Kolkman and Kelly, 2003; Miklas, 2007; Soule et al., 2011; Miklas et al., 2013). Molecular markers developed for this QTL were used for marker assisted selection (MAS) in common bean breeding programs (Miklas, 2007; Ender et al., 2008).

Given the importance of this major meta-QTL WM2.2 in WM resistance, narrowing the interval, investigating additional QTL effects, and identifying candidate genes will provide a basic understanding of the genetic mechanisms of resistance to this disease. The results obtained from this study should enable development of tightly linked markers leading towards the molecular characterization and functional validation of the causative gene and assisting breeding and improvement of WM resistance in common bean.

## Material and methods

### Plant materials and phenotyping

Two populations, R31, consisting of 105 F_5:7_ RILs, and Z0726–9, consisting of 86 F_5:7_ RILs, were used for a WM2.2 QTL-based sequencing study (**Supplementary Figure S1**). R31 was generated from a cross between ‘Raven’ and ‘I9365–31’, two black bean genotypes. Raven is known to be a highly susceptible cultivar to WM disease (Kelly et al., 1994), while I9365–31 is a partially resistant germplasm line derived from an interspecific cross between *P. vulgaris* and *P. coccineus* (Miklas et al., 1998). The resistant genetic factors within I9365–31 is presumably from the *P. coccineus* resistant parent since it possesses high levels of resistance to WM and has multiple WM meta-QTL (Schwartz et al., 2006; Soule et al., 2011; Vasconcellos et al., 2017). Besides WM2.2, QTL on Pv04, Pv05, Pv06, Pv07, and Pv08 were also reported in R31 population (Soule et al., 2011).

The Z0726–9 population was derived from a cross between ‘AN–37’ and ‘PS02–029C–20’. AN–37, a RIL selected from the Aztec/ND88 RIL population, was released as the WM resistant pinto bean USPT-WM–1 (Miklas et al., 2014). AN37 is the putative source of the WM2.2 QTL and possesses WM3.1 resistance QTL as well (Miklas et al., 2007). The PS02–029C–20 (Matterhorn*4/NY6020–4) parent was reported to possess the WM8.3 QTL but not the WM2.2 QTL (Mamidi et al., 2016).

The field reaction to WM disease was evaluated at physiological maturity as described by Miklas et al. (2001), based on a 1 to 9 scale where 1 is no disease development, and 9 is completely dead plants. The phenotypic data for the Z0726–9 population was collected during the 2009 and 2010 growing seasons. The mean 2009 field data was obtained from two ratings a few weeks apart with three replications, and the mean of 2010 field data was scored only once across three replications. The phenotypic data for the R31 population was initially published in Soule et al. (2011). The data was collected during 2000 and 2001 growing seasons, with three replications. The score for the individual plant within the plot with the most disease was recorded as well. In addition, the field score used for the QTL analysis was adjusted using plant stand as a covariate. Mean phenotypic scores combining two years data for both populations were used for three independent QTL mapping analyses (Classic, bulked segregant analysis, and Khufu QTL-seq).

### Classic QTL mapping

To confirm the existence of the WM2.2 QTL in both populations, a linkage map was re-generated using the SNP and SSR markers of Pv02 chromosome (Vasconcellos et al., 2017) and additional Insertion-Deletion (InDel) markers developed within the physical boundaries of meta-QTL WM2.2. The InDels were designed through assembly and alignment of the sequence data from two Durango race genotypes, USPT-WM–1 [WM2.2 resistance, (Miklas et al., 2014)] and Matterhorn [WM2.2 susceptible, (Kelly et al., 1999)]), against the *P. vulgaris* v2.1 reference genome sequence (https://phytozome-next.jgi.doe.gov/info/Pvulgaris_v2_1) (Schmutz et al., 2014). By comparing the mapped sequences of the two genotypes in IGV 2.3 (Robinson et al., 2011), a total of 48 InDel markers were generated within the 3 to 23 Mb interval on Pv02 with a minimum size of 6 bp and a mean of 19.7 bp. The PCR protocol used to amplify all InDel markers was: 3 min at 95°C for one cycle, 20 s at 95°C, 30 s at 55°C, and 1 min at 72°C for 45 cycles, and 10 min at 72°C for one cycle. For population R31, all Pv02 SNPs and InDels were used to generate a linkage map, but for population Z0726–9, only InDel markers were employed. Mapdisto v2.1.8.7 (Heffelfinger et al., 2017) was used to generate linkage groups based on a minimum logarithm of odds (LOD) score of 5.5 and maximum recombination frequency of 30%. QTL analysis for both populations were performed by composite interval mapping (CIM) using the *cim ()* function with the r/qtl package (Broman et al., 2003). CIM was implemented using the Haley–Knott (hk) regression method with a window size of 5 cM and selecting 1 marker as covariate. The LOD value was calculated based on 1000 permutation tests at the *α* = 0.05 experiment-wide error rate (Churchill and Doerge, 1994). The significant QTL boundary was based on genetic intervals that passed the threshold of LOD score (2.35 for R31 and 1.63 for Z0726–9). Percentage of phenotypic variation (PVE) explained by each QTL, and average additive effects were calculated using the *fitqtl ()* function implemented in R/qtl. The confidence interval for identified QTL was calculated using the 95% Bayes interval method implementing *bayesint ()* function. Markers within the WM2.2 meta-QTL were further used to identify resistant and susceptible genotypes for bulked DNA sequencing.

### Bulk segregant QTL analysis based on fixed SNPs

Genotypes with extreme WM resistant or susceptible phenotypes, and possessing alleles associated with those phenotypes based on the QTL analysis, were selected for bulking. DNAs of the selected extreme lines from R31 and Z0726–9, along with their resistant parental lines, were extracted using MagMax magnetic bead purification protocol (https://www.thermofisher.com). The resistant and susceptible DNA bulks, along with the resistant parental lines, were sequenced (2x150 bp paired-end reads on Illumina X10) at HudsonAlpha Institute for Biotechnology (https://hudsonalpha.org/gsc/).

For each population, poor-quality bases were trimmed using Sickle (Joshi and Fass, 2011). Paired-end reads were aligned against the resistant parent sequence using BWA-MEM (Li, 2013). The resistant parental reference genome was developed from *P. vulgaris* reference genome, G19833 v2.1 using “Fasta Alternate Reference Maker” algorithms in GATK v3.3 (McKenna et al., 2010). After mapping, the Picard MarkDuplicates was used to remove duplicates for each of the bulks. Sequences were indexed using Samtools (Li et al., 2009). SNP variants for the susceptible bulk for each population were called using GATK Unified Genotyper v3.3 with a minimum confidence threshold of 30 and filtered for a minimum read depth of five.

For each pool, we calculated the reference allele frequency as the ratio of reads that matched the reference allele to the total number of reads at that particular position using in-house scripts (https://github.com/sujanmamidi/Pop-Seq). The SNPs were categorized into fixed (the reference allele frequency is 100% in one bulk and 0% in the other bulk), shared (polymorphic in both bulks, the reference allele frequency is between 0 and 100), unique-resistant (polymorphic in only the resistant pool, but one allele is fixed in susceptible bulk), or unique-susceptible (polymorphic in only the susceptible pool, but one allele is fixed in resistant bulk). The count of fixed SNPs for 10kb window with a 2kb slide (10kb–2kb) was performed. The significant regions of the genome were estimated at 95% and 99% percentile tail using 10,000 bootstraps.

### Validation of WM2.2 QTL by Khufu *de novo* QTL-seq

To validate and further narrow the WM2.2 QTL interval in both populations, we next performed Khufu *de novo* QTL-seq (hereafter regarding it as QTL-seq), a newly developed pipeline by HudsonAlpha for mapping complex traits (Korani et al., 2021). Resistant and susceptible DNA bulk sequences for the R31 and Z0726-9 populations were subjected to the Khufu pipeline and mapped to common bean reference genome G19833 v2.1 (https://phytozome-next.jgi.doe.gov/info/Pvulgaris_v2_1). QTL-seq analysis was performed on each population to identify the differences in allele frequencies between each bulk. The difference in allele frequency is represented by the variable deltaVAR and is normalized between 0 and 1, where 0 indicates an equal frequency between bulks, and 1 indicates complete segregation between bulks. The identified variants of the resistant and susceptible bulk (same RILs as bulked DNA sequencing) corresponding to the WM2.2 QTL were used to generate input R object (rds) which was created as an output of the Khufu analysis pipeline. To visualize the peak genomic regions, the rds R object was uploaded into a shiny app v3.0 (https://w-korani.shinyapps.io/khufu_var3/) by selecting rawSNP type using the following threshold: MAF 0.40, Min depth 50 (40 for Z0726-9), Max depth 5000, interval 1000, and window size 100. Areas of the genome with a difference in allele frequency (i.e. deltaVAR ~ 0.75 to 1) indicate QTL regions for the phenotypic difference between the two bulks.

### Candidate genes

Candidate genes were selected within the physical boundaries of the detected peak genomic regions of the G19833 v2.1 genome. Peak genomic interval, determined by considering the overlapping genomic interval of the identified QTL or peak region by three independent QTL mapping analyses, was used as the physical interval to scan for putative candidate genes associated with WM resistance. In addition, the potential effect (missense, nonsense, etc.) of fixed SNPs within candidate gene sequence was obtained using snpEFF (Cingolani et al., 2012).

## Results

### Phenotype

**Figure 1:**
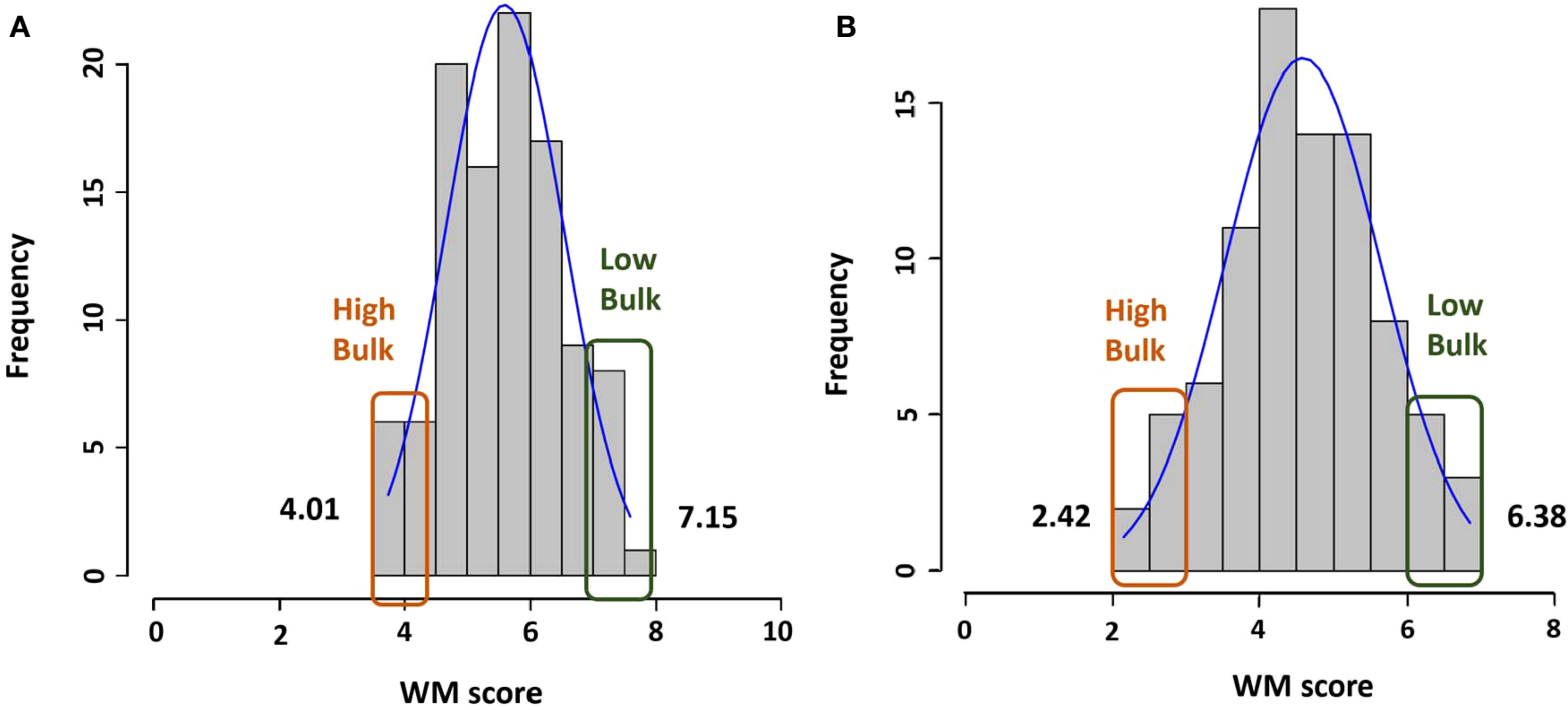
Histogram of WM phenotype scores for all lines and selected lines with the highest and lowest scores in **(A)** R31 and **(B)** Z0726-9 population.

The mean WM score for population R31 was 5.6 with a range of 3.7 to 7.6, and for Z0726–9, the mean was 4.6 with a range of 2.2 to 7.3 (**Figure 1**). Phenotypic values for both populations were normally distributed (KS test *P*-value at *α*_0.01 = _1.63), and the WM score mean between the resistant (score < 5 for R31 and < 4 for Z0726–9) and susceptible (score > 6 for R31 and > 5 for Z0726–9) haplotypes were significant (**Table 1**). Z0726–9 lines with resistant haplotypes showed significantly lower (more resistant) WM scores compared with R31 lines, which likely results from the presence of an additional QTL (WM8.3) contributed by the parent PS02–029C–20 (Mamidi et al., 2016).

**Table 1:** Phenotypic means and t-test values for two populations.

Population		Number	WM score mean
R31	Resistant WM2.2 haplotype	32	4.5
	All other lines	73	6.07
	t-test		6.91 × 10^-27^
	Susceptible WM2.2 haplotype	36	6.63
	All other lines	69	5.05
	t-test		1.86 × 10^-26^
	Resistant WM2.2 haplotype	32	4.5
	Susceptible WM2.2 haplotype	36	6.63
	t-test		9.14 × 10^-31^
Z0726-9	Resistant WM2.2 haplotype	24	3.34
	All other lines	62	5.06
	t-test		2.11 × 10^-17^
	Susceptible WM2.2 haplotype	31	5.68
	All other lines	55	3.9
	t-test		2.97 × 10^-20^
	Resistant WM2.2 haplotype	24	3.34
	Susceptible WM2.2 haplotype	31	5.06
	t-test		2.11 × 10^-17^

### QTL mapping

**Figure 2:**
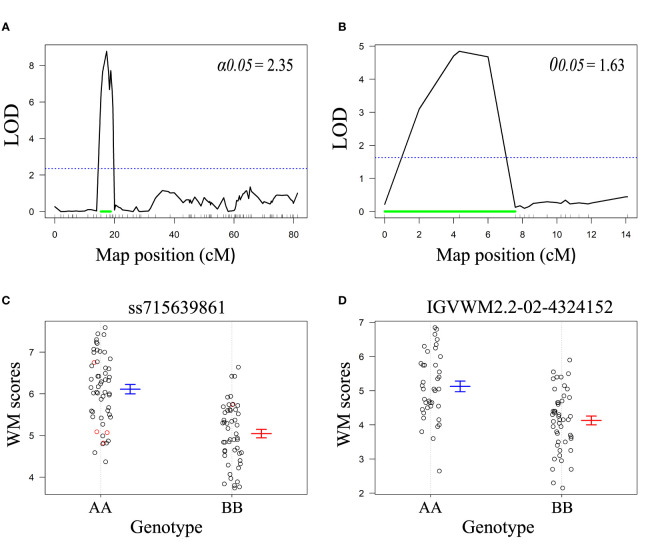
LOD profiles for WM2.2 QTL using composite interval mapping in **(A)** R31 and **(B)** Z0726-9 population. The blue dotted horizontal line represents LOD threshold at 5% level of significance after 1000 permutations. The green horizontal line indicates 95% the 95% Bayes intervals of the identified QTL. Genotypic effects of peak markers **(C)** ss715639861 detected in R31, and **(D)** IGVWM2.2-02-4324152 detected in Z0726-9 population. The ‘A’ allele was derived from the susceptible parent and ‘B’ allele was derived from the resistant parent in both populations.

Classic QTL analysis confirmed the existence of WM2.2 in both populations (**Figure 2**). For R31, WM2.2 was detected within an interval of 15.4 to 18.7 cM, corresponding to a physical interval of 3.87-11.30 Mb (peak marker ss715648338 at 4.54 Mb) on Pv02 with the LOD value of 8.8 (**Figures 2A, C**). In Z0726–9, the QTL was detected within the genomic interval of 3.57 to 10.40 Mb on Pv02 at a LOD value of 4.9 (**Figures 2B, D**). The phenotypic variation explained by the identified QTL in R31 and Z0726-9 population were 32% and 22.9%, respectively. The additive effects were –0.53 for R31 and –0.50 for Z0726–9, respectively, implying that resistant parents contributed beneficial allele for lower disease scores (**Supplementary Table S1**).

For R31, the SNP alleles from five markers including: ss715647659, 3.58 Mb; ss715648336, 4.28 Mb; ss715648335, 4.33 Mb; ss715639861, 4.48 Mb; and ss715648338, 4.54 Mb, were used to select genotypes for resistant *vs* susceptible bulks (**Supplementary Table S2**). For Z0726–9, ten InDel markers: WM2.2–02– 3566770 (bp), WM2.2–02– 4324152, WM2.2–02– 6411447, WM2.2–02–10403733, WM2.2–02–10425994, WM2.2–02–10436002, WM2.2–02–12724443, WM2.2–02–13107477, WM2.2–02–14728780 and WM2.2–02–16830328 were used to define genotypes for bulking (**Supplementary Table S2**).

### Selection of bulks

Based on both phenotypic data and WM 2.2 marker alleles, two extreme bulks for each population were generated. DNA of 33 genotypes (R31: resistant n = 8, susceptible n = 9; Z0726–9: resistant n = 8, susceptible n =6; plus two resistance parents) were extracted and pooled accordingly (**Table 2**). The mean of resistant and susceptible bulks for R31 were respectively 4.0 (with a range of 3.7 to 4.3) and 7.1 (with a range of 6.8 to 7.4). The mean values of the two bulks are significantly different (*p*-value = 9.14 × 10^–31^). For Z0726–9, the mean of resistant and susceptible bulks were respectively 2.4 (with a range of 1.9 to 2.8) and 6.4 (with a range of 6.1 to 6.7). The mean values of the two bulks are significantly different (*p*-value = 2.11 × 10^–17^).

**Table 2:** Summary of WM score for selected lines used for resistant and susceptible bulks.

	R31		Z0726-9	
	Resistant bulk	Susceptible bulk	Resistant bulk	Susceptible bulk
No. of lines	8	9	8	6
Mean	4.01	7.07	2.43	6.38
Range	3.74-4.32	6.77-7.42	1.9-2.8	6.1-6.7

### Sequencing, variant detection, and QTL identification by bulk-segregant analysis (BSA)

For the R31 population, 223 million reads (~ 55x) were obtained from the resistant parent I9365–31, and 95.71% of these were mapped. For this population, 257 million reads for susceptible and 240 million reads for resistant bulks, were generated (**Supplementary Table 3**). For the Z0726–9 population, 180 million reads (~45x) were obtained for the resistant AN–37 parent and 95.67% of these were mapped. A total of 239 million reads for susceptible and 204 million reads for resistant bulks were obtained for this population. The resistant parent from each population served as reference-based parental genome sequence. Therefore, reads for each bulk were mapped to the appropriate parental genome sequence. After filtering, we identified ~197k SNPs for the R31 bulks and ~278k SNPs for the Z0726–9 bulks.

**Figure 3:**
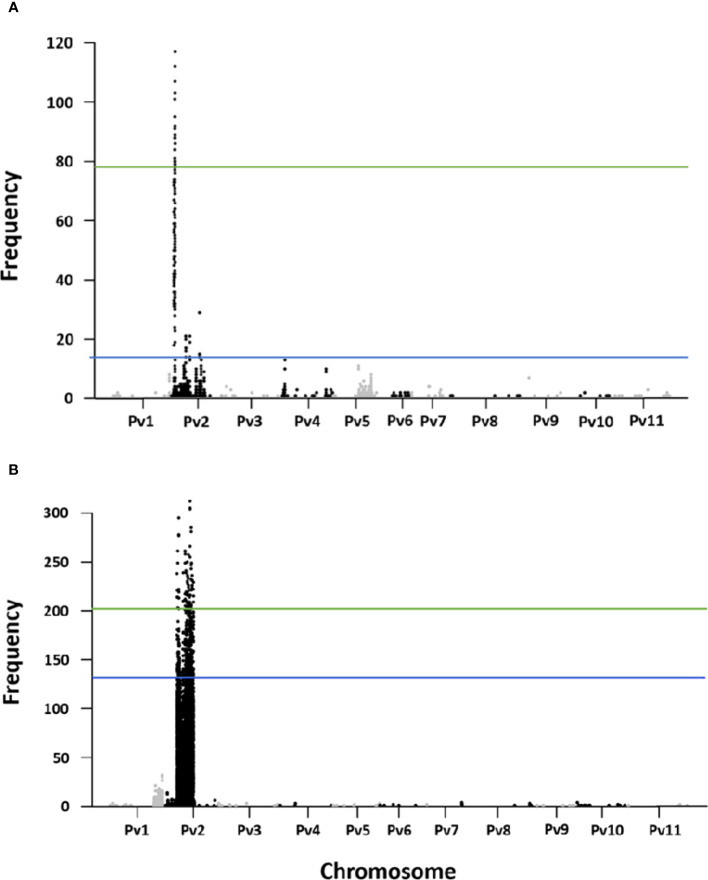
Fixed SNP counts in 10k-2k windows in **(A)** R31, and **(B)** Z0726-9 population.

A total of 2,414 fixed SNP sites were detected between the resistant and susceptible pools for the R31 population. Two QTL based on the significant windows (at 99 percentile) were identified in the euchromatic regions positioned at Pv02:3.54–3.56 Mb (with an average of 68 SNP per 10k window) and Pv02:4.27–4.56 Mb (with an average 82 SNP per 10k window). The fixed SNP peak was located at 3.54 and 4.48 Mb respectively (**Figure 3A**). For the Z0726–9 population, there were 66,189 fixed SNPs between the pools. For this population, nine QTL based on the significant windows (at 99 percentile) were identified in the heterochromatic region of 12.19 to 26.41 Mb on Pv02. Fixed SNP peaks were located at 12.20, 12.99, 13.58, 17.45, 19.14, 19.93, 21, 23.38, and 26.32 Mb respectively in each window. The highest number of SNPs (233) was located at 13.83–13.92 Mb significant window (**Figure 3B**).

### Genomic regions identification using Khufu *de novo* QTL-seq

**Figure 4:**
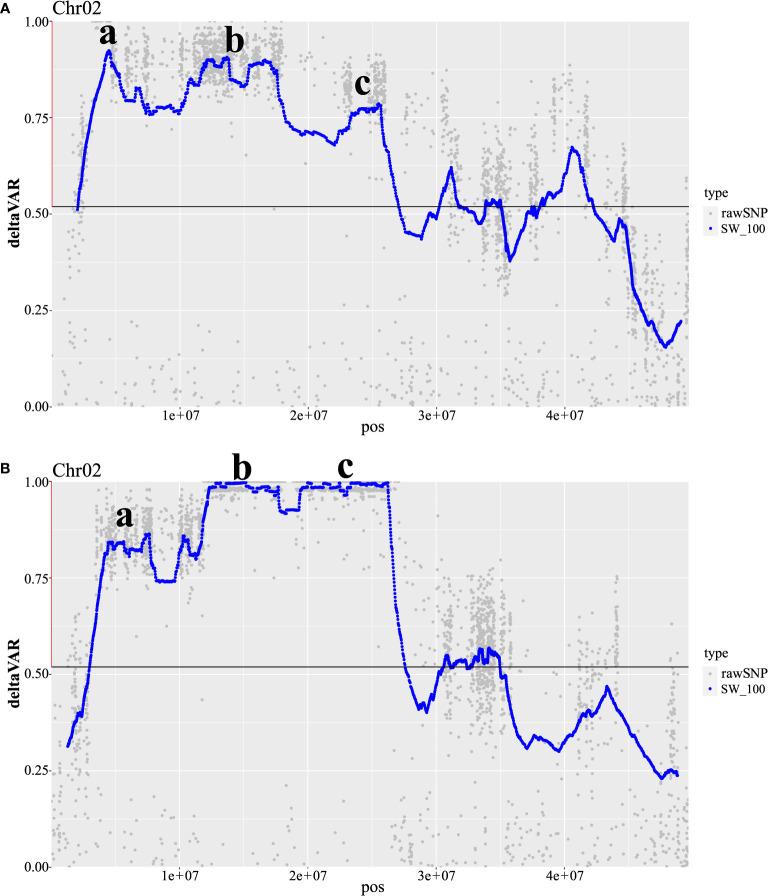
Khufu *de novo* QTL-seq analysis for the validation and identification of additional genomic regions in the meta-QTL WM2.2 interval in R31 and Z0726-9 RIL populations. deltaVAR—the difference in allele frequency between the resistant bulk and the susceptible bulk is 0 to 1, where 1 is resistant allele. **(A)** In R31, the first major peak **(a)** is identified within the 4.16-5.76 Mb (deltaVAR ~ 0.90), second peak **(b)** is detected within 11.86-17.67 Mb (deltaVAR ~ 0.89), and third peak **(c)** is located within 23.01-25.74 Mb (deltaVAR ~ 0.77) physical interval on Pv02. **(B)** In Z0726-9, the first major peak **(a)** is identified within the 4.27-7.69 Mb (deltaVAR ~ 0.86), second peak **(b)** is detected within 12.19-17.61 Mb (deltaVAR ~ 0.99), and third peak **(c)** is located within 19.44-26.29 Mb (deltaVAR ~ 0.99) on Pv02 overlapped with the R31 identified regions associated with white mold resistance.

Our efforts to validate the presence of WM2.2 QTL using the newly developed Khufu *de novo* QTL-seq pipeline successfully identified WM2.2 QTL in both populations. This mapping approach yielded three distinctive peaks on Pv02 in R31 and Z0726-9 population (**Figure 4**). The peak genomic regions in R31 are located within the 4.16-5.76 Mb (deltaVAR ~ 0.90), 11.86-17.67 Mb (deltaVAR ~0.89), and 23.01-25.74 Mb (deltaVAR ~0.77) interval (**Figure 4A**); whereas 4.27-7.69 Mb (deltaVAR ~0.86), 12.19-17.61 Mb (deltaVAR ~0.99), and 19.44-26.29 Mb (deltaVAR ~0.99) are the peak regions detected on Pv02 in Z0726-9 population (**Figure 4B**). The first strong peak detected by QTL-seq in R31 and Z0726-9 population were consistent with the detected QTL interval in both populations by classic QTL mapping and bulk segregant analysis (only in R31) (**Figures 2**–**4**). This further confirms the presence of WM2.2 QTL in the ~ 3.54-5.76 Mb genomic interval. Moreover, an additional peak was revealed by QTL-seq, located within the 11.86-17.68 Mb and 12.19-17.61 Mb on Pv02 in the R31 and Z0726-9, respectively, indicating the presence of an additional region of interest. This region overlapped within the identified genomic interval spanning 12.19 to 26.41 Mb in Z0726–9 population by BSA (**Figures 2**–**4**). Moreover, QTL-seq also detected peaks on Pv02 located between 23.01-25.74 Mb in R31 and 19.44-26.29 Mb interval in Z0726-9 population. The interval for this third WM2.2 QTL is also within the “genomic” interval (12.19-26.41 Mb) for Z0726-9 population that was revealed by BSA (**Figures 3**, **4**). The identified QTL/peak region by three independent QTL mapping analyses in R31 and Z0726-9 population were summarized in **Table 3**.

**Table 3:** Summary of the identified QTL/peak genomic regions by three independent QTL mapping analysis in R31 and Z0726-9 population.

		Physical interval of peak genomic regions (Mb)			
QTL	Population	Classic QTL mapping ^a^	Bulk segregant analysis	Khufu *de novo* QTL-seq	Significant region ^b^
WM2.2a	R31	3.87-11.30 PVE: 32%; LOD: 8.8	3.54-4.56	4.16-5.76	4.27-5.76
	Z0726-9	3.57-10.40 PVE: 22.9%; LOD: 4.9	Not detected	4.27-7.69	
WM2.2b	R31	Not detected	Not detected	11.86-17.67	12.19-17.61
	Z0726-9	Not detected	12.19-26.41	12.19-17.61	
WM2.2c	R31	Not detected	Not detected	23.01-25.74	23.01-25.74
	Z0726-9	Not detected	12.19-26.41	19.44-26.29	

Classic QTL mapping ^a^: PVE, phenotypic variation explained; LOD, logarithm of the odds.

Significant region ^b^: Significant region of each QTL was determined by considering the overlapping genomic interval of the identified QTL or peak region by three independent QTL mapping analyses.

Candidate genes

For the WM2.2a region (4.27-5.76 Mb) defined in the R31 and Z0726-9 population, five gene models were identified as candidates based on the G19833 v2.1 reference genome (**Table 4**). Of these, gene models (Phvul.002G049000, Phvul.002G052600, Phvul.002G053300, Phvul.002G055600, and Phvul.002G055700) encoded an ortholog of gibberellin 2-oxidase 8, leucine-rich repeat (LRR) family protein, pentatricopeptide repeat (PPR) superfamily protein, receptor-like protein kinase 1, and ethylene responsive element binding factor 1 proteins, respectively. SNPs within each of these gene models have a potential modifier effect on WM2.2a resistance. For the WM2.2b detected in both R31 and Z0726–9 population, which has a much broader interval overlapped within 12.19–17.61 Mb, six gene models were identified as candidates (**Table 4**). Nine gene models encoding pathogenesis-related thaumatin superfamily protein (2 copies), leucine-rich receptor-like protein kinase family protein, leucine-rich repeat (LRR) family protein, basic chitinase (2 copies), and protein kinase superfamily protein (2 copies) were discovered as putative candidate genes within the WM2.2c QTL interval (23.01-25.74 Mb) (**Table 4**). Clusters of LRR family proteins and pathogenesis-related thaumatin superfamily proteins in the R31 and Z0726–9 population were described to have a modifier effect. A domain analysis of the LRR proteins in this interval revealed that only gene model Phvul.002G079200, an ortholog of TIR-NBS-LRR class of disease resistance proteins, has all the required NB-ARC domain amino acid signatures (GKTTL, GLPL, and MHD) necessary for the activation of a functional disease resistance gene (Sukarta et al., 2016).

**Table 4:** Candidate genes identified in the significant WM2.2 QTL windows.

Population and QTL	Significant region ^a^	G19833 gene model	Physical position (bp)	*Arabidopsis* gene model	Gene	*Arabidopsis thaliana* define
**R31 and Z0726-9 (WM2.2a)**	4.27-5.67	Phvul.002G049000	4,566,992- 4,572,915	AT4G21200.1	ATGA2OX8	gibberellin 2-oxidase 8
		Phvul.002G052600	4,793,413-4,795,331	AT3G43740.1		Leucine-rich repeat (LRR) family protein
		Phvul.002G053300	4,857,194-4,860,054	AT5G02860.1		Pentatricopeptide repeat (PPR) superfamily protein
		Phvul.002G055600	5,726,005-5,728,416	AT5G60900.1	RLK1	receptor-like protein kinase 1
		Phvul.002G055700	5,761,710-5,763,021	AT4G17500.1	ATERF-1	ethylene responsive element binding factor 1
**R31 and Z0726-9 (WM2.2b)**	12.19-17.61	Phvul.002G079000	12,278,025-12,279,434	AT1G69970.2	CLE26	CLAVATA3/ESR-RELATED 26
		Phvul.002G079200	12,311,752-12,319,211	AT1G27170.1		transmembrane receptors;ATP binding (TIR-NBS-LRR CLASS DISEASE RESISTANCE PROTEIN-RELATED)
		Phvul.002G079700	12,383,017-12,385,510	AT5G23400.1		Leucine-rich repeat (LRR) family protein
		Phvul.002G083000	13,147,125-13,154,633	AT2G04030.1	AtHsp90	Chaperone protein htpG family protein
		Phvul.002G089300	15,224,493-15,227,775	AT1G14390.1		Leucine-rich repeat protein kinase family protein
		Phvul.002G094600	17,479,531-17,480,874	AT1G15000.1	scpl50	serine carboxypeptidase-like 50
**R31 and Z0726-9 (WM2.2c)**	23.01-25.74	Phvul.002G107800	23,024,776-23,026,523	AT4G38660.1		Pathogenesis-related thaumatin superfamily protein
		Phvul.002G107900	23,056,171-23,058,495	AT4G36010.2		Pathogenesis-related thaumatin superfamily protein
		Phvul.002G111100	23,785,218-23,789,905	AT4G36180.1		Leucine-rich receptor-like protein kinase family protein
		Phvul.002G112100	24,049,894-24,051,147	AT5G66330.1		Leucine-rich repeat (LRR) family protein
		Phvul.002G114100	24,422,348-24,424,101	AT3G12500.1	ATHCHIB	basic chitinase
		Phvul.002G114200	24,446,860-24,447,980	AT3G12500.1	ATHCHIB	basic chitinase
		Phvul.002G115700	24,770,509-24,771,096	AT1G64160.1		Disease resistance-responsive (dirigent-like protein) family protein
		Phvul.002G115800	24,775,122-24,777,863	AT2G17220.1		Protein kinase superfamily protein
		Phvul.002G116200	24,837,381-24,840,429	AT2G39360.1		Protein kinase superfamily protein

Significant region ^a^: Significant region of each QTL was determined by considering the overlapping genomic interval of the identified QTL or peak region by three independent QTL mapping analyses.

### Non-synonymous fixed sites

The scan of non-synonymous fixed sites using snpEff identified additional regions associated with WM disease in each population. For R31, gene models Phvul.002G046600, Phvul.002G048200 and Phvul.002G048900 [respectively orthologs of heat shock protein (HSP70), protodermal factor (PDF) and tyrosyl-DNA phosphodiesterase-related (TDP)] and for Z0726–9, gene model Phvul.002G114500 ortholog of squamosa promoter binding protein (SPL) were annotated as non-synonymous fixed sites with moderate effects on WM (**Supplementary Table S4**). Among these gene models associated with missense variants, the role of HSP70 in plant immune response has been studied extensively.

## Discussion

Advances in high throughput sequencing technologies and statistical tools enable the mapping of genetic factors associated with complex traits and the subsequent identification of candidate genes (McClean et al., 2018; Oladzad et al., 2019). Next generation sequencing, NGS, is widely utilized for QTL and GWAS (genome wide association studies) analyses to identify the regions responsible for a specific phenotype. Both QTL and GWAS methods have advantages and disadvantages. Bi-parental QTL analysis can identify a strong genetic factor, but the confidence interval is larger due to fewer recombination events in the population. GWAS utilizes a high number of recombination events within the population leading to the identification of narrower QTL interval compared to biparental QTL mapping. However, association mapping studies are constrained because many QTL may be observed that explain only a small portion of the phenotypic variation. To overcome the limitations of QTL mapping and association mapping, and to narrow the interval of an important QTL for WM resistance in common bean, we used bulked-segregant analysis (BSA) coupled with marker selection (Mamidi et al., 2016). To optimize the BSA, we used marker data from a preliminary biparental QTL analysis along with phenotypic data to select lines for the resistant and susceptible DNA bulks. Two RIL populations which possess WM resistance from different genetic backgrounds were evaluated. Given the WM2.2 intervals were large in these populations, it was of interest to narrow this QTL using a combination of classic QTL mapping and BSA approaches. Moreover, we validated the existence of WM2.2 meta-QTL and identified additional QTL influencing the effects of meta-QTL WM2.2 using Khufu *de novo* QTL-seq pipeline.

The R31 WM QTL was mapped to 3.87-11.30 Mb and 3.54-4.56 Mb interval based on classic QTL mapping and significant fixed SNP windows, respectively within the interval of meta-QTL WM2.2, while Z0276–9 WM QTL was also mapped to an overlapping interval spanning 4.32 to 10.40 Mb by classic QTL mapping on Pv02. Khufu also successfully detected the same overlapping genomic region (4.16 to 5.76 Mb in R31 and 4.27 to 7.69 Mb in Z0726-9) from bulk DNA sequences. Based on the overlapping intervals resulting from the three independent mapping approaches and both populations, this region (WM2.2a) was delimited to 4.27 to 5.76 Mb interval on WM2.2 meta-QTL. This region also overlaps with the detected QTL in the O83 (Orion//Orion/R31-83), AN (Aztec/ND88-106-04), and R31 populations (Vasconcellos et al., 2017). The phenotypic variation explained by the WM2.2a QTL were 32 and 23% for R31 and Z0276–9 population, respectively consistent with the previous findings (Soule et al., 2011; Vasconcellos et al., 2017). This indicates WM2.2a QTL has a major effect and is an ideal candidate for improving WM resistance in common bean using MAS because it appears consistently in multiple genetic backgrounds.

QTL-seq detected two distinctive peaks within the meta-QTL WM2.2 interval from R31 and Z0726-9 bulk DNA sequences, indicating the presence of additional QTL in both populations which was not revealed by classic QTL mapping. QTL-seq identified strong peaks in R31 positioned at 11.86-17.67 Mb and 23.01-25.74 Mb regions that overlapped with two strong peak genomic regions (12.19-17.61 Mb and 19.44-26.29 Mb) detected in Z0726-9 population, which was identified as a single region (12.19-26.24 Mb) by BSA. The genomic intervals for these identified regions are relatively large compared to the WM2.2a region. These large intervals likely resulted from the low level of recombination in the Pv02 heterochromatic region that is estimated to extend from 8.0–27.1 Mb in the G19833 v2.1 reference genome of common bean. This has been seen previously in common bean where the WM8.3 QTL was identified in the heterochromatic region of Pv08 across 10.75 Mb, while QTL WM7.1 mapped in the euchromatic region of Pv07 to a narrow 1.25 Mb interval (Mamidi et al., 2016). In another comprehensive study of 771 QTL related to 161 unique sorghum traits, the heterochromatic region had a mean QTL density of 11QTL/0.5 Mb compared to 7.5 QTL/0.5 Mb in the euchromatic region (Mace and Jordan, 2011). Although QTL detected in the recombination-poor regions might complicate successful gene cloning, they can be very useful in MAS breeding programs. The detection of additional genomic regions by QTL-seq and BSA indicates the possibility of two additional associated genomic regions corresponding to the WM2.2 meta-QTL interval that might influence the resistance response upon *S. sclerotiorum* infection. Therefore, combining multiple approaches of QTL mapping with a greater number of markers and sequence-based introgression, it was determined that the meta-QTL WM2.2 consists of three independent QTL regions, WM2.2a (1.49 Mb interval), WM2.2b (5.42 Mb interval), and WM2.2c (2.73 Mb interval). WM2.2a physically mapped in the euchromatic region (4.27-5.76 Mb); WM2.2b and WM2.2c in the heterochromatic regions 12.19-17.61 Mb and 23.01-25.74 Mb, respectively, may provide different WM tolerance/resistance mechanisms. Meta-QTL analysis for WM resistance detected WM2.2 QTL in four mapping populations and was physically mapped within 3.11-3.57 Mb (straw test) in O83 (Orion//Orion/R31-83); 3.91-5.02 Mb (field test) in AN (Aztec/ND88-106-04); 4.55-21.76 Mb (field test) in R31 (Raven/I9365-31), and 12.54-23.21 (straw and field) Mb in BV (Benton/VA19) population (Vasconcellos et al., 2017). These results support our identification of three independent QTL within the 3.11 to 23.21 Mb physical position on Pv02. A recent GWAS of WM resistance in snap bean germplasm identified significant SNPs located on 34.9, 41.8, and 51.3 Mb on Pv02 (Arkwazee et al., 2022). However, none of the identified SNPs were found to overlap with our reported genomic interval. The dry bean QTLs in Pv02 are uniquely localized and different relative to those found in snap bean germplasm. Moreover, use of multiple methods to map WM QTL in the overlapped genomic interval in different genetic backgrounds and narrow down the QTL interval. This validates the effects of these QTL leading a step forward towards the development of functional markers and provides a basis for fine mapping and cloning of causative genes conferring WM resistance.

Our fine mapping revealed potential candidate genes associated with synonymous and/or non-synonymous fixed sites in WM2.2a, WM2.2b, and WM2.2c intervals. Pentatricopeptide repeat (PPR) proteins and gibberellin 2-oxidase are two important disease tolerance/resistance associated gene models detected in WM2.2a. PPR proteins share sequence features with NLR disease resistance genes particularly for their involvement in diversifying selection process. It has been shown that the birth and death process described for immunoglobulin genes (Nei et al., 1997), and later for plant R genes (Michelmore and Meyers, 1998) is also the route for tandem repeat duplications in the PPR protein gene family (Geddy and Brown, 2007). Many studies have shown that PPR proteins contain many sequence-specific RNA binding sites involved in several RNA processing activities (Saha et al., 2007; Fujii and Small, 2011; Laluk et al., 2011; Yagi et al., 2014; Schaper and Anisimova, 2015). For example, the mRNA encoding PPR proteins are the target for miR400 that down regulates the PPR genes by cleaving their mRNAs and subsequently the plant becomes more susceptible to pathogenetic bacteria and fungi (Park et al., 2014). Moreover, recent studies on sequenced genomes of several plants revealed that the nucleotide binding site of PPR protein genes are similar to the NB-ARC nucleotide binding site of NLR genes which are part of defense mechanism used by plants to respond to pathogen infection (Sekhwal et al., 2015).

Gibberellin 2-oxidase is a candidate gene that may explain the WM disease avoidance mechanism associated with plant architecture. Gibberellins controls inter-node length and therefore indirectly the shape of the bean plant canopy. Shorter internode plants have an upright canopy that allows airflow through the field which in turns reduces the high humidity environment favored by the WM pathogen. Long internode plants become prostrate on the ground which leads to a low, dense canopy with high humidity conditions favored by the pathogen. This leads to an increased severity of WM disease in the field (Miklas et al., 2013; Tivoli et al., 2013). Similarly, plants with larger internodes were found to be more susceptible to *S. sclerotiorum* infection under field conditions in canola (Roy et al., 2021). Gibberellin 2 oxidase inactivates gibberellins and effectively changes the status of this important hormone for internode extension. In soybean, copy number increase of gibberellin 2-oxidase 8 genes showed reduced shoot length and suppression of trailing growth by decreasing the internode length that corresponds to reduced lodging by reducing the level of bioactive gibberellins in the plants apical bud (Wang et al., 2021). This feature makes gibberellin 2-oxidase an important candidate gene because WM2.2 is associated with canopy porosity, canopy height, and lodging in the R31 population. Vasconcellos et al. (2017) detected a QTL for canopy porosity, collocated (5.8 Mb) with WM2.2a in R31 and Z0726-9 population. This further supports avoidance related genes as primary candidates. Moreover, significant fixed SNP sites and Khufu detected in the WM2.2a interval were located inside the start site of the gibberellin 2-oxidase gene which makes it a reasonable candidate gene for disease avoidance traits.

Hsp70, another candidate gene in the WM2.2a interval, interacts with other plant defense genes (Lee et al., 2012). HSPs are molecular chaperon proteins which play an important role in modulating the structure of disease resistance proteins and modulate Arabidopsis defense against pathogens (Noeül et al., 2007). In another study, Jelenska et al. (2010) reported that the *P. syringae* pathogen uses the HopI1 effector to hijack Hsp70-associated chaperoning activity. Under heat stress conditions, if excess HSP70 is provided, this effector is dispensable for *P. syringae* pathogenicity. This suggests searches for candidate resistance genes should look beyond NB-ARC genes and consider other genes which encode proteins that could be a target of a pathogen effector. These other genes/proteins may be critical cellular proteins that directly or indirectly influence R-gene functions by modulating their structure or/and stability following their interaction with an effector (Su et al., 2018).

The significant WM2.2b interval discovered in the R31 and Z0726–9 populations were identified in the heterochromatic region. A cluster of six LRR encoding genes (Phvul.002G079200, Phvul.002G079700, Phvul.002G081300, Phvul.002G089300, Phvul.002G092200, Phvul.002G092300), typical of plant disease associated resistance genes, were detected in this region (12.19-17.61 Mb). This finding suggests a role in physiological resistance for WM2.2b QTL. Partial physiological resistance is important when the severity of the disease overcomes the avoidance mechanisms present. However, when all of the LRR clusters in this region (19.78–20.21 Mb) were analyzed in Pfam (Bateman et al., 2004), only a single TIR-NBS-LRR gene model (Phvul.002G079200; 12.31 Mb) possessed all NB-ARC domain, typical of one class of functional disease resistance protein. This class of protein plays an important role in regulating plant disease resistance pathways and is a reasonable candidate gene to consider within the WM2.2b QTL.

A pathogenesis-related thaumatin superfamily protein candidate gene is located within the 23.01-25.74 Mb interval of WM2.2c. The antifungal activity of thaumatin like proteins (TLP) gene was found to provide protection against different fungal pathogens. *ObTLP1* showed antifungal activity under *in vitro* conditions, and ecotypic expression of *ObTLP1* in Arabidopsis led to enhanced tolerance against *S. sclerotiorum* and *Botrytis cinerea* (Misra et al., 2016). A candidate gene that encodes a disease resistance-responsive (dirigent-like protein) family protein is also found in the WM2.2c interval. This gene family is involved in lignan biosynthesis. In response to pathogen infection, lignin deposition is considered to function as an effective physical barrier to pathogen attacks (Moerschbacher et al., 1990).

## Conclusions

Using additional markers for classical QTL analysis, fixed sites for bulked segregant QTL analysis, and Khufu *de novo* QTL-seq, the WM 2.2 QTL was fine-mapped into three separate regions, WM2.2a (4.27-5.67 Mb), WM 2.2b (12.19 to 17.61 Mb), and WM2.2c (23.01-25.74 Mb). QTL WM2.2a may trigger an avoidance mechanism, possibly through gibberellin 2-oxidase. When confronted with moderate to severe WM disease in the field, WM2.2b may induce physiological resistance via a traditional NLR reaction network. WM2.2c QTL may provide resistance to plants by producing pathogenesis related proteins through the induction of systematic acquired resistance mechanisms. The same QTL-linked marker haplotypes used to select lines for the contrasting bulks and nonsynonymous SNPs identified by candidate gene analysis (**Tables S2**, **S4**) provide a starting point for the marker assisted selection of the three WM2.2 QTL to combine avoidance and physiological resistance to white mold disease in common bean germplasm. Our future efforts will be directed to the design of functional genetic markers, validating their effects for genomics-assisted MAS breeding and functional validation of the candidate genes to improve WM resistance in dry bean.

## Data availability statement

The original contributions presented in the study are included in the article/**Supplementary Files**. Further inquiries can be directed to the corresponding author/s.

## Author contributions

AO, JR, SM, PEM, and PNM designed and conceived the experiment. AO and JR wrote the manuscript. PNM performed experiments in the field and greenhouse, developed the populations, provided the phenotyping and linkage mapping data for the classic QTL analysis. RL designed and ran the InDel markers. JC , ZM and WK performed the formal analysis of Khufu *de novo* QTL-seq. AO, JR, SM, and PEM discussed the results and interpretation of the final data and PNM provided suggestions to improve it. All authors contributed to the article and approved the submitted version.
