# Supplementary material for: Linked candidate genes of different functions for white mold resistance in common bean (Phaseolus vulgaris L) are identified by multiple QTL mapping approaches
Source: Front Plant Sci. 2023 Jul 31;14:1233285. doi: 10.3389/fpls.2023.1233285 (PMC10425182; doi:10.3389/fpls.2023.1233285)
Supplement: Supplementary file 2 [file Presentation_1.pptx]

## Slide 1
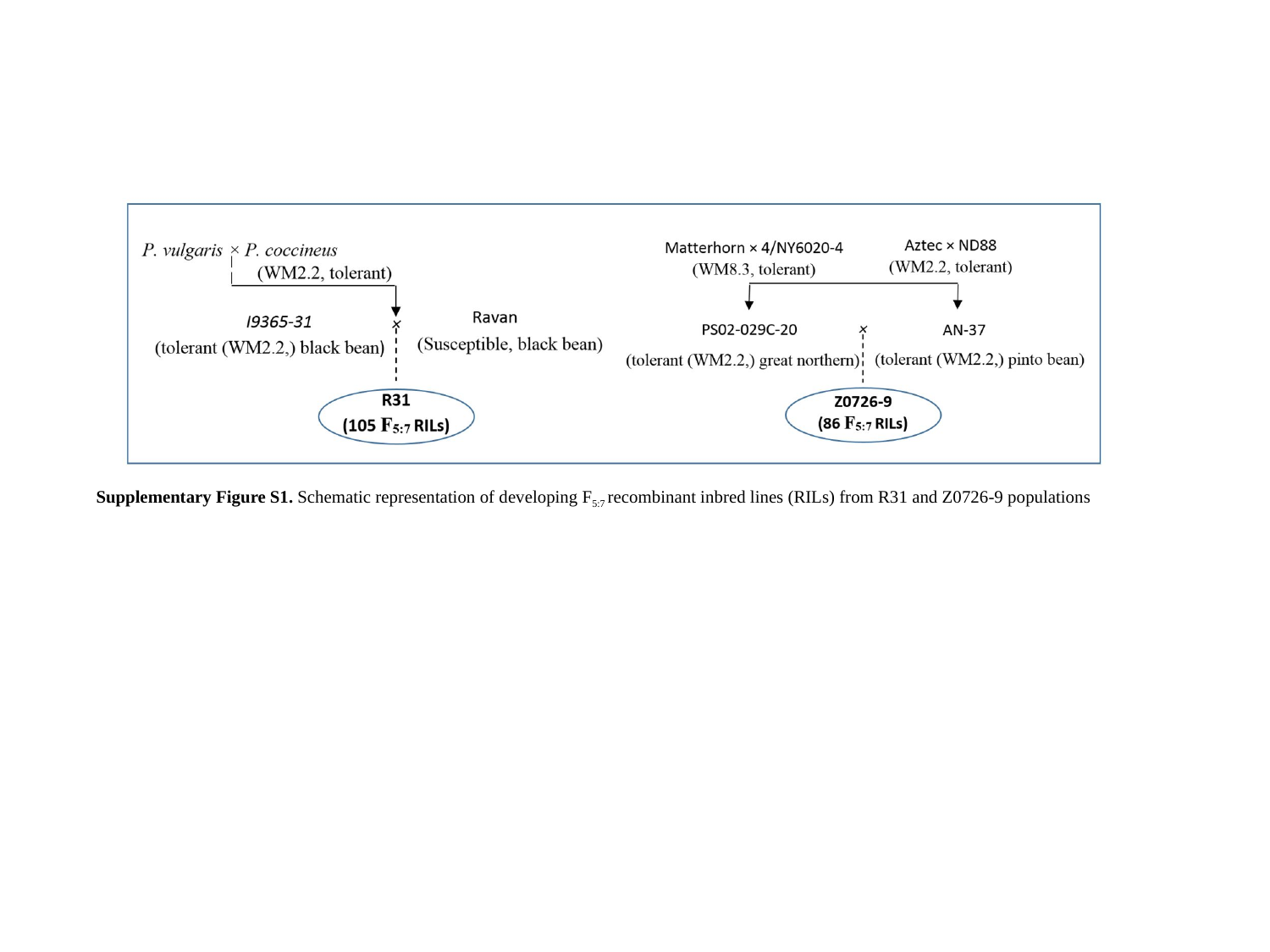

Supplementary Figure S1. Schematic representation of developing F5:7 recombinant inbred lines (RILs) from R31 and Z0726-9 populations
